# The Role of Social Media in Enhancing Diabetes Knowledge and Health Behaviors: A Cross-Sectional Study

**DOI:** 10.7759/cureus.87608

**Published:** 2025-07-09

**Authors:** Ohud M Alshamrani, Najlaa M Alsudairy, Saad M Alsudairy

**Affiliations:** 1 Primary Health Care, King Abdulaziz Medical City, National Guard Hospital, Jeddah, SAU; 2 Obstetrics and Gynaecology, Maternity and Children Specialized Hospital, Jeddah, SAU

**Keywords:** chronic disease management, diabetes education, diabetes knowledge, diabetes mellitus, digital health, health behavior, health communication, public health, saudi arabia, social media

## Abstract

Background: Diabetes mellitus is a major public health concern worldwide, with high prevalence in Saudi Arabia. Traditional diabetes education methods have limited reach, especially among younger populations. Social media platforms are increasingly used for health information, but their impact on diabetes knowledge and behavior in Saudi Arabia is not well understood.

Methods: We conducted a cross-sectional study from February to April 2025 involving 430 adults (≥18 years) across Saudi Arabia, recruited via social media using snowball sampling. Participants completed a questionnaire assessing demographics, social media use related to diabetes, diabetes knowledge (five-item score), and self-reported behavior changes. Multivariable linear regression identified predictors of knowledge scores.

Results: Among 430 respondents, 217 (50.5%) were female, 124 (28.8%) were aged 25-34 years, 131 (30.5%) had diabetes, and 60 (14.0%) had healthcare education. Daily social media use for diabetes content was reported by 121 participants (28.1%). The mean diabetes knowledge score was 3.84 ± 1.13 (out of 5), with daily users scoring higher (4.19 ± 0.88, n=121) than rare users (3.52 ± 1.28, n=102) or non-users (2.96 ± 1.35, n=39) (P < 0.001). Behavioral changes due to social media were reported by 268 participants (62.4%). In adjusted models, daily social media use (β=0.55; 95%CI, 0.38 to 0.73; P < 0.001), healthcare education (β=0.42; 95%CI, 0.26 to 0.59; P < 0.001), and having diabetes (β=0.36; 95%CI, 0.19 to 0.53; P < 0.001) were independently associated with higher knowledge scores. Increasing age was inversely associated with knowledge (β=-0.07 per decade; 95%CI, -0.12 to -0.01; P=0.018).

Conclusions: Frequent social media engagement with diabetes content is independently associated with greater diabetes knowledge and self-reported behavior change among adults in Saudi Arabia. Integrating social media into diabetes education strategies may enhance public health efforts in regions with high disease burden, provided content accuracy is ensured through clinical oversight.

## Introduction

Diabetes mellitus remains one of the most pressing public health challenges globally, affecting an estimated 537 million adults as of 2021, a number projected to rise to 783 million by 2045. The burden is particularly pronounced in the Middle East and North Africa region, where prevalence rates are among the highest worldwide [1-3]. In Saudi Arabia, recent estimates indicate that over 18% of the adult population is living with diabetes, with an even greater proportion at risk due to obesity, sedentary lifestyle, and poor dietary habits [3,4]. As the disease burden continues to escalate, effective strategies for improving public knowledge, prevention, and self-management are urgently needed.

Traditional models of diabetes education delivered through clinic-based sessions, printed materials, or structured programs have shown efficacy but remain limited in reach, scalability, and engagement, particularly among younger populations. The advent of digital technology, especially social media platforms, has transformed the landscape of health communication [2,4]. Platforms such as YouTube (Google LLC, Mountain View, California, United States), Instagram (Meta Platforms, Inc., Menlo Park, California, United States), TikTok (ByteDance Ltd, Haidian, Beijing, China), and X (Bastrop, Texas, United States) have emerged as major sources of health information, offering rapid, visually engaging, and often peer-driven content. These platforms are widely used in Saudi Arabia, particularly among individuals under 40 years of age, presenting a unique opportunity for public health outreach. However, the quality, accuracy, and impact of diabetes-related content on these platforms remain poorly understood [2-7].

While prior studies have explored the role of social media in chronic disease management, few have quantified its effect on diabetes-related knowledge, behavior, or educational outcomes, particularly in non-Western settings [8,9]. Furthermore, concerns persist regarding misinformation, variable source credibility, and the potential for lay audiences to act on content without professional guidance [10-12]. Understanding how social media influences diabetes literacy is critical for designing effective digital health strategies in high-burden regions.

This study aimed to assess the association between social media use and diabetes-related knowledge among adults in Saudi Arabia. We hypothesized that greater exposure to diabetes-related content on social media would be associated with higher knowledge scores and a greater likelihood of self-reported behavior change.

## Materials and methods

Study design and setting

This was a cross-sectional, questionnaire-based study conducted between February and April 2025 in Saudi Arabia. The study aimed to assess the impact of social media and online platforms on diabetes-related knowledge, attitudes, and behaviors among adults. The survey was distributed electronically using a secure, online data collection platform (Google Forms; Google LLC), allowing access across all regions of the country.

Participants

Eligible participants were adults aged 18 years or older, residing in Saudi Arabia, and able to read Arabic or English. Individuals were recruited through social media platforms (including WhatsApp (Meta Platforms, Inc.), Twitter/X, Instagram, and Telegram (Telegram Messenger Inc., Tortola, British Virgin Islands) using a snowball sampling technique. Healthcare professionals and laypersons were both included. Participation was voluntary and anonymous, and electronic informed consent was obtained before survey initiation.

Survey instrument

The structured questionnaire was developed in English and translated into Arabic using a forward-backward translation methodology to ensure accuracy and conceptual equivalence. The final survey included 20 multiple-choice, closed-ended questions divided into five sections: demographics and clinical characteristics (e.g., age, gender, education, diabetes status); social media usage patterns related to diabetes content; knowledge of diabetes, measured using five factual multiple-choice questions based on international guidelines; behavioral influences of social media on health actions; and perceived educational benefit of social media content. All knowledge questions had a single correct answer. A cumulative knowledge score (range 0-5) was calculated by summing the number of correct responses.

Validity and pilot testing

The draft questionnaire was reviewed by three experts in endocrinology, public health, and digital health for content validity. It was piloted on a sample of 20 respondents to assess clarity, response time, and technical functionality. Minor modifications were made based on feedback. Data from the pilot were excluded from the final analysis.

Outcomes

The primary outcome was the association between the frequency of social media use and diabetes knowledge score. Secondary outcomes included the influence of social media on self-reported behavioral changes and perceived knowledge gain.

Statistical analysis

Descriptive statistics were used to summarize participant characteristics and response patterns. Categorical variables were presented as frequencies and percentages, while continuous variables were reported as means with standard deviations. Group comparisons of knowledge scores by frequency of social media use were conducted using one-way analysis of variance (ANOVA). A multivariable linear regression model was constructed to identify independent predictors of diabetes knowledge score, including age, gender, education level, healthcare background, diabetes status, and social media use frequency. All statistical analyses were performed using IBM SPSS Statistics for Windows, version 29.0 (Released 2022; IBM Corp., Armonk, New York, United States). A two-sided P value of <0.05 was considered statistically significant.

## Results

Participant characteristics

A total of 430 respondents completed the survey (Table 1). The largest age group was 25-34 years (n=124; 28.8%). Gender distribution was balanced, with 217 (50.5%) identifying as female. Most participants were Saudi nationals (n=361; 83.9%). Nearly half held a bachelor’s degree (n=207; 48.1%), and 60 (14.0%) had healthcare-related education. A total of 131 participants (30.5%) reported having diabetes, including 37 (8.6%) with type 1 diabetes and 94 (21.9%) with type 2 diabetes. Most respondents (n=311; 72.3%) reported a family history of diabetes.

**Table 1:** Demographic and clinical characteristics of respondents (N = 430)

Characteristics		Frequency (Percentage)
Age group (years)	18–24	96 (22.3%)
	25–34	124 (28.8%)
	35–44	89 (20.7%)
	45–54	72 (16.7%)
	55+	49 (11.4%)
Gender	Male	213 (49.5%)
	Female	217 (50.5%)
Nationality	Saudi	361 (83.9%)
	Non-Saudi (Arab)	41 (9.5%)
	Non-Saudi (Non-Arab)	28 (6.5%)
Education Level	High school or below	74 (17.2%)
	Bachelor’s degree	207 (48.1%)
	Master’s or PhD	89 (20.7%)
	Healthcare-related education	60 (14.0%)
Has diabetes	Yes, Type 1	37 (8.6%)
	Yes, Type 2	94 (21.9%)
	No	279 (64.9%)
	Not sure	20 (4.7%)
Family history of diabetes	Yes	311 (72.3%)
	No	98 (22.8%)
	Not sure	21 (4.9%)

Social media use for diabetes information

Social media use for health-related content was common. Of the 430 participants, 121 (28.1%) reported using it daily, and 167 (38.8%) used it a few times per week (Table 2). YouTube was the most frequently used platform for diabetes-related content (n=108; 25.1%), followed by Instagram (n=97; 22.6%) and TikTok (n=79; 18.4%). A total of 262 participants (60.9%) reported seeing general diabetes information, 219 (50.9%) saw nutrition-related content, and 188 (43.7%) saw posts that included medical advice from professionals.

**Table 2:** Social media usage patterns related to diabetes (N = 430) Note: Participants could select more than one type of content.

Variables		Frequency (Percentage)
Frequency of using social media for health info	Daily	121 (28.1%)
	Few times per week	167 (38.8%)
	Rarely	89 (20.7%)
	Never	53 (12.3%)
Platform used most for diabetes info	YouTube	108 (25.1%)
	Instagram	97 (22.6%)
	TikTok	79 (18.4%)
	X (formerly Twitter)	54 (12.6%)
	Facebook	32 (7.4%)
	Do not follow diabetes content	60 (14.0%)
Type of content seen	General information	262 (60.9%)
	Nutrition tips	219 (50.9%)
	Exercise guidance	173 (40.2%)
	Personal stories	115 (26.7%)
	Medical advice from professionals	188 (43.7%)

Diabetes knowledge

Overall knowledge was moderately high (Table 3). The most correctly answered question was on diabetes symptoms, with 369 (85.8%) correctly identifying increased thirst, followed by knowledge of appropriate foods for diabetes (n=348; 80.9%) and the cause of type 2 diabetes (n=331; 77.0%). The mean total knowledge score was 3.84 (SD ±1.13) out of 5.

**Table 3:** Diabetes knowledge scores based on social media usage (N = 430) This table summarizes the frequency and percentage of respondents who correctly answered each of the five diabetes knowledge questions. Correct options were based on international clinical guidelines. The mean total knowledge score (range 0–5) was calculated by summing correct responses for each participant (3.84 ± 1.13).

Knowledge Question (Correct Option)	Frequency (Percentage)
Common symptom (Increased thirst)	369 (85.8%)
The main cause of Type 2 diabetes (Insulin resistance)	331 (77.0%)
Fasting blood glucose (70–100 mg/dL)	309 (71.9%)
Diabetic-safe food (Grilled chicken and vegetables)	348 (80.9%)
True insulin statement (May be needed if orals fail)	293 (68.1%)

Behavioral impact of social media

Most participants reported that social media influenced their behavior (Table 4). Of the 430 respondents, 122 (28.4%) reported changing their behavior multiple times due to social media exposure, and 146 (34.0%) did so once or twice. A total of 173 participants (40.2%) had shared diabetes-related content, and 150 (34.8%) reported following advice from social media without consulting a healthcare provider. This included 41 (9.5%) who did so often and 109 (25.3%) who did so occasionally.

**Table 4:** Impact of social media on diabetes-related behaviors (N = 430) This table presents self-reported behavioral responses to diabetes-related content seen on social media. It includes the frequency of behavior changes attributed to social media exposure, whether participants shared diabetes-related content, and whether they followed advice without consulting a healthcare professional.

Behavior/Perception		Frequency (Percentage)
Changed behavior due to social media	Yes, multiple times	122 (28.4%)
	Yes, once or twice	146 (34.0%)
	No	138 (32.1%)
	Not applicable	24 (5.6%)
Shared diabetes-related content	Yes	173 (40.2%)
	No	239 (55.6%)
	Don't remember	18 (4.2%)
Followed advice without doctor consultation	Yes, often	41 (9.5%)
	Yes, once or twice	109 (25.3%)
	No	258 (60.0%)
	Don't remember	22 (5.1%)

Perceived educational benefit

A majority of participants perceived social media as helpful for diabetes education (Table 5). Of the 430 respondents, 139 (32.3%) reported that social media significantly improved their diabetes knowledge, and 189 (44.0%) reported some improvement. A total of 253 respondents (58.8%) said they would recommend diabetes-related content on social media to others.

**Table 5:** Perceived impact of social media on knowledge and education (N=430) This table summarizes participants’ perceptions of how social media affected their diabetes-related knowledge and their willingness to recommend such content to others. Responses reflect self-assessed educational benefit and perceived value of the content.

Measure	Response	Frequency (Percentage)
Perceived Improvement in Knowledge	Significant	139 (32.3)
	Somewhat	189 (44.0)
	Slight	72 (16.7)
	Not at all	30 (7.0)
Would Recommend Diabetes Content	Yes	253 (58.8)
	Maybe	93 (21.6)
	No	51 (11.9)
	Not sure	33 (7.7)

Association between social media use and knowledge

Knowledge scores increased with the frequency of social media use for health purposes (Table 6). The mean knowledge score was highest among daily users (4.19 ± 0.88), compared with those who used social media rarely (3.52 ± 1.28) or never (2.96 ± 1.35). This association was statistically significant (P < 0.001 by one-way ANOVA).

**Table 6:** Association between social media use frequency and diabetes knowledge score (N=430) This table displays the mean diabetes knowledge scores (± standard deviation) stratified by frequency of social media use for health information. A statistically significant association was observed using one-way analysis of variance (ANOVA), F(3, 426) = 28.45, P < 0.001.

Frequency of Use	Knowledge Score, mean ± SD	P-value (ANOVA)
Daily	4.19 ± 0.88	< 0.001
Few times per week	3.93 ± 1.02	
Rarely	3.52 ± 1.28	
Never	2.96 ± 1.35	

Predictors of knowledge score

In multivariable linear regression (Table 7), daily social media use was significantly associated with higher diabetes knowledge scores (β = 0.55; 95%CI, 0.38 to 0.73; P < 0.001). Other independent predictors included healthcare-related education (β = 0.42; 95%CI, 0.26 to 0.59; P < 0.001) and diabetes (β = 0.36; 95%CI, 0.19 to 0.53; P < 0.001). Increasing age was associated with a slight reduction in knowledge (β = -0.07 per decade; 95%CI, -0.12 to -0.01; P = 0.018). Female gender was not a significant predictor (P = 0.148).

**Table 7:** Multivariable linear regression: predictors of knowledge score This table presents the β coefficients, 95% confidence intervals (CI), and P-values for variables independently associated with diabetes knowledge scores. Statistically significant predictors are indicated by P-values < 0.05.

Variable	β Coefficient	95% CI	P-value
Age (per decade)	-0.07	(-0.12, -0.01)	0.018
Healthcare-related education	+0.42	(0.26, 0.59)	<0.001
Daily social media use	+0.55	(0.38, 0.73)	<0.001
Female gender	+0.11	(-0.04, 0.26)	0.148
Has diabetes	+0.36	(0.19, 0.53)	<0.001

## Discussion

In this cross-sectional survey of 430 adults in Saudi Arabia, we found that social media use was significantly associated with higher levels of diabetes-related knowledge. Participants who used social media platforms more frequently for health content, particularly daily, scored higher on a standardized diabetes knowledge questionnaire. In addition, a substantial proportion of respondents reported behavior change and perceived knowledge improvement resulting from exposure to diabetes-related content on social media. These findings highlight the growing influence of digital platforms on public health education in the context of chronic diseases such as diabetes.

Our results are consistent with prior research demonstrating the role of social media as a health information source and behavior change facilitator. Notably, our study also confirms concerns raised in earlier literature regarding the potential for individuals to act on unverified online content [10-16]. More than one-third of our participants acknowledged following diabetes-related advice from social media without medical consultation. 

The significant association between social media use and knowledge scores suggests that platforms such as YouTube, Instagram, and TikTok may be valuable tools in augmenting public diabetes education, especially when traditional healthcare access is limited [7,13]. These platforms can serve as effective outreach channels for health authorities, provided the content is accurate, evidence-based, and delivered by credible sources.

Importantly, the findings indicate a high level of receptivity to social media health content among young adults and those without formal medical training. This presents an opportunity to strategically deploy tailored content that addresses common misconceptions-particularly around insulin use and dietary management promote evidence-based guidance. The widespread sharing behavior observed in our sample (40.2%) further amplifies the potential reach of well-crafted digital health messages.

Strengths and limitations

This study has several strengths. It is one of the first to quantitatively assess the impact of social media exposure on diabetes knowledge in a Middle Eastern population using a validated, structured questionnaire. The relatively large and demographically diverse sample enhances the generalizability of the findings within Saudi Arabia. Moreover, the inclusion of a multivariable regression model allows for the adjustment of potential confounders such as age, gender, and healthcare education.

However, the study also has limitations. The use of a self-administered online survey may have introduced selection bias, favoring younger, digitally literate individuals. The cross-sectional design precludes causal inference, and self-reported behavior changes could be subject to recall or social desirability bias. Furthermore, the knowledge score, while grounded in evidence-based questions, assessed only selected domains of diabetes literacy and may not fully capture clinical decision-making capacity. Finally, although we measured exposure to diabetes content on social media, we did not assess the quality or source credibility of that content, which may vary widely across platforms.

## Conclusions

In this cross-sectional study, frequent use of social media platforms was independently associated with higher levels of diabetes-related knowledge and self-reported behavior change among adults in Saudi Arabia. These findings highlight the potential of social media as a complementary tool for public health education in regions with a high burden of diabetes. However, they also underscore the importance of ensuring that digital health content is evidence-based and professionally curated to mitigate misinformation. Integrating social media into national diabetes education strategies, while maintaining clinical oversight, may offer a scalable and culturally relevant approach to improving public literacy and self-care practices. Future longitudinal studies are needed to assess the durability of these effects and their translation into clinical outcomes.
